# Increased Dietary Inflammatory Index Is Associated with Schizophrenia: Results of a Case–Control Study from Bahrain

**DOI:** 10.3390/nu11081867

**Published:** 2019-08-11

**Authors:** Haitham Jahrami, Mo’ez Al-Islam Faris, Hadeel Ali Ghazzawi, Zahra Saif, Layla Habib, Nitin Shivappa, James R. Hébert

**Affiliations:** 1 Ministry of Health, Manama, Bahrain; 2 College of Medicine and Medical Sciences, Arabian Gulf University, Manama, Bahrain; 3 Department of Clinical Nutrition and Dietetics, College of Health Sciences/Research Institute of Medical and Health Sciences (RIMHS), University of Sharjah, P. O. Box 27272 Sharjah, UAE; 4 Department of Nutrition and Food Technology, Faculty of Agriculture, The University of Jordan, Amman, Jordan; 5 Cancer Prevention and Control Program, University of South Carolina, Columbia, SC 29208, USA; 6 Department of Epidemiology and Biostatistics, Arnold School of Public Health, University of South Carolina, Columbia, SC 29208, USA; 7 Connecting Health Innovations LLC, Columbia, SC 29201, USA

**Keywords:** dietary inflammatory index (DII), schizophrenia, case–control study

## Abstract

Background: Several studies have indicated that chronic low-grade inflammation is associated with the development of schizophrenia. Given the role of diet in modulating inflammatory markers, excessive caloric intake and increased consumption of pro-inflammatory components such as calorie-dense, nutrient-sparse foods may contribute toward increased rates of schizophrenia. This study aimed to examine the association between dietary inflammation, as measured by the dietary inflammatory index (DII^®^), and schizophrenia. Methods: A total of 120 cases attending the out-patient department in the Psychiatric Hospital/Bahrain were recruited, along with 120 healthy controls matched on age and sex. The energy-adjusted DII (E-DII) was computed based on dietary intake assessed using a comprehensive food frequency questionnaire (FFQ). Logistic regression was used to estimate odds ratios and 95% confidence intervals, adjusting for potential confounders including age, sex, body mass index, education, employment, diabetes, hypertension, and cardiovascular disease with E-DII expressed both as a continuous variable and categorized as quartiles. Results: The mean E-DII score for the entire sample was 1.79 ± 1.52, indicating a generally pro-inflammatory diet. The cases with schizophrenia appeared to have a higher E-DII score compared to controls: 1.99 ± 1.39 vs. 1.60 ± 1.38, respectively (*p* = 0.009). For every one unit increase in the E-DII score, the odds of having schizophrenia increased by 62% (OR 1.62; 95% CI 1.17–2.26). Similarly, increased risk was observed when the E-DII was used as quartiles, with participants in most pro-inflammatory quartile 4 being nearly 6 times more likely to be schizophrenic than participants in the most anti-inflammatory group quartile 1 (OR 5.96; 1.74–20.38; *p*-trend = 0.01). Conclusions: The data suggest that a pro-inflammatory diet, as indicated by increasing E-DII score, is associated with schizophrenia. This is the first study to examine the association between the DII and schizophrenia in a Middle Eastern population. Although these results are consistent with findings from research conducted in depression, additional studies are required before generalizing the findings to other populations.

## 1. Introduction

Patients with schizophrenia die approximately 20 years earlier than individuals in the general population, even after controlling for the risk of suicide and accidents [1]. The vast majority of early deaths in patients with schizophrenia are attributable to preventable medical conditions, namely cardiovascular disease (CVD) and chronic obstructive pulmonary disease (COPD), metabolic syndrome, type 2 diabetes (T2DM), and some types of cancer [2,3,4,5,6]. Recent research shows that dietary components are recognized to exert various effects on inflammation and their links with many diseases [7]. Therefore, having a comprehensive understanding of modifiable risk factors and their link with inflammation is essential to mitigate or prevent premature mortality.

Low-grade systemic inflammation is characterized by the continuous presence of pro-inflammatory cytokines through increased blood flow during injury, which is known to play an essential role in the development of illnesses and related mortality [8]. Several meta-analyses have shown that increased risk of mortality (e.g., CVD, cancers, and COPD) is associated with higher intake of pro-inflammatory processed food [5,8,9]. A large body of evidence supports the role of inflammation in the pathophysiology of mental health disorders [10,11,12]. For example, the literature on the DII and depression is sufficiently robust to have produced one meta-analyses, and many other studies have been published recently on the topic [13,14,15,16,17,18,19,20,21]. Environmental factors have been found to substantially impact the pathogenesis of psychiatric disorders including schizophrenia [22,23,24,25], with dietary patterns having been shown to modulate the inflammatory state, thus, highlighting their potential role as an etiologic factor or therapeutic tool in disorders with an inflammatory basis [9,17,26,27]. Indeed, a recent review indicates that diet-associated inflammation may be associated with severe mental illness, including schizophrenia [8].

For patients with schizophrenia, psychotropic medications are essential components of management. Many of these medications are associated with significant weight gain, central obesity, and development of metabolic disorders. A recent meta-analysis suggests that one of the key factors underlying these cardiometabolic abnormalities are the effects of antipsychotic medications on excessive energy intake and poor diet quality [28]. Patients on anti-psychotic medications report increased appetite and increased cravings for sweet foods and beverages, and reduced intake of healthy foods such as vegetables and fruits [29,30]. This craving makes them more susceptible to consuming a diet with increased inflammatory potential that will worsen their condition.

The Dietary Inflammatory Index (DII^®^) is a novel literature-derived, population-based index developed to assess the inflammatory potential of a person’s diet [31]. Its purpose is to evaluate the quality of diet based on its inflammatory potential. The index places the individual on a continuum from maximally anti-inflammatory diet to maximally pro-inflammatory diet. The DII was based on the literature on six inflammatory markers C-reactive protein (CRP), interleukin (IL)-6, IL-1β, IL-10, IL-4, and Tumor Necrosis Factor (TNF) α [31] related to any dietary factor. A high DII score represents a more pro-inflammatory diet, whereas lower DII scores represent a more anti-inflammatory diet [31]. The DII has been shown to be validated with a variety of inflammatory markers including CRP, IL-6, TNF, homocysteine and others in several studies [32,33,34,35]. With respect to mental health disorders, the DII has been shown to be associated with depression [19,20], anxiety [27], cognitive decline [36,37], and severe mental disorders, including schizophrenia [8,9].

This study is the first to assess the association between the inflammatory potential of foods consumed by patients with schizophrenia (as measured through the DII) in comparison to age- and sex-matched controls in a Middle Eastern population.

## 2. Materials and Methods

The guidelines of the Strengthening the Reporting of Observational Studies in Epidemiology (STROBE) Statement) were adopted in planning, implementing, and reporting the study [38].

### 2.1. Setting

Recruitment of cases with schizophrenia was carried in the Psychiatric Hospital, Ministry of Health, Kingdom of Bahrain. The Psychiatric Hospital is the national center for diagnosis, treatment, and rehabilitation of patients with severe mental illness in the nation of Bahrain. Data were collected from patients between March and December 2016.

### 2.2. Sample Size and Sampling Procedure

The sample size was estimated for this study using a projected sample size for a matched case–control study. This was based on an asymptotic z test, with a 1:1 ratio of matched design assuming an OR = 2.0 of exposure in cases relative to controls. The sample size was estimated for the two-sided test with error probabilities of α = 0.05 and 80% power (β = 0.20). Approximately 100 cases were necessary for powered analysis; thus, we included 120 cases to further power our research. Sample size calculation was performed using Stata 15.0 (StataCorp LLC., College Station, TX, USA). The sample of schizophrenia cases was selected using simple random sampling technique from the case registry.

### 2.3. Participants

This study was performed using a case–control design. Cases with schizophrenia (N = 120) were included based on these inclusion criteria: Either sex, aged between 20–60 years, diagnosed with any schizophrenia of any type according to the International Statistical Classification of Diseases 10^th^ Revision, and were attending the out-patients department (OPD). Patients who were pregnant/lactating, who were dieting, or were participating in lifestyles interventions or clinical trials were excluded from the study. The control group was selected randomly from the primary health care centers and based on matching the demographic characteristics, including age (by exact year of birth) and sex. The exclusion criteria for the control group were the presence of a positive history of psychiatric illness, a serious medical condition (e.g., cancer, SLE), being pregnant/lactating women, and those who had been dieting during the past six months. The ratio of recruitment was 1:1; thus, for each case, one control was enrolled. A simple randomization technique was used after matching to select the right control. The majority of the population in the OPD are with three to eight years of history with schizophrenia.

### 2.4. Ethical Consideration

Ethical approval for this study was obtained from the Research Ethics Committee, Ministry of Health, Kingdom of Bahrain (according to the meeting number 02/2016). Written informed consent was sought and obtained from the participants before collecting data. Participation in this study was entirely voluntary, with no monetary or non-monetary incentives.

### 2.5. Assessments and Data Collection

All of the participants were interviewed in order to collect the required information for the study. During the interview, anthropometric measurements (body weight and height) also were taken. Digital scales were used to measure participant’s weight, with a height rod attachment that was kept on a firm horizontal surface. Weight was recorded to the nearest 0.1 kg with the participants wearing light clothes and not wearing shoes. The height was measured with the rod attached to the weighing scale to the nearest 1.0 cm, and body mass index (BMI, kg/m^2^) was calculated from measured weight and height. The BMI was then categorized as per the World Health Organization (WHO) categories of underweight, normal, overweight, or obese.

The questionnaire comprised of demographics, medical history, psychiatric history for the cases, and selected lifestyle factors including smoking status, physical activity level, and sleep. Dietary intake was assessed using a semi-quantitative food frequency questionnaire (FFQ) [29]. The FFQ was used to assess the frequency of food consumption in the study sample. Participants were asked to report their usual frequency of consumption, on average, during the past month that they had consumed a standard serving of a specific food item. The frequency response was categorized into six categories (1 time/day, ≥2 times/day, 1–2 times/week, 3–6 times/week, 1–3 times/month, rarely or never). Each food was specified in standardized serving size, and portion size photographs and geometrical models were used to clarify the serving sizes for vegetables, meat, rice, milk, and other food items. See Supplementary 1, the research questionnaire. 

### 2.6. DII Score

Dietary intake assessed using the FFQ were analyzed using ESHA Nutrition and Fitness software (ESHA Food Processor SQL, version 10.1.1, Salem, OR, USA). These nutrients were analyzed for each subject recruited into the study. As ESHA software was developed for Western countries, Bahraini traditional and conventional single and composite foods and dishes not included in ESHA database were created and introduced into the database, either by formulating the recipe for composite dishes using the available ingredients in the software, or inserting the nutritional value for that single food or composite dish as obtained from local food composition tables, and then introducing it into the ESHA database. The original DII consists of 45 nutrient and nutritional factors. However, most studies that use an FFQ for dietary assessment cannot account for all of the components. A complete description of the process of developing the DII has been published elsewhere [31,39]. Briefly, the dietary data of the sample was first linked to the world database that provided a robust estimate of a mean and SD for each parameter [31]. This was achieved by subtracting the “standard global mean” from the intake reported via the FFQ and dividing this value by the standard deviation (SD) to obtain “z” scores. To minimize the effect of “right skewing,” these “z” scores were then converted to a centered proportion. The centered proportion for each food parameter for each individual was then multiplied by the respective food parameter effect score (inflammatory potential for each food parameter), which was derived from the literature review, to obtain a food parameter-specific DII score for an individual. All of the food parameter–specific DII scores were then summed to create the overall DII score for each participant in the study [31]. Finally, Energy-adjusted DII (E-DII) scores were calculated using the density method wherein all food parameters were converted to per 1000kcal of nutrients and the same procedure was used to relate individual exposure data to the global energy-adjusted database. In the present study, the available components that were used for calculating E-DII included the following nutrients: Energy, carbohydrates, fiber, protein, fat, saturated fatty acids (SFAs), polyunsaturated fatty acids (PUFAs), monounsaturated fatty acids (MUFAs), omega-3 fats, omega-6 fats, saturated fat, cholesterol, trans-fat, vitamins B_12_, B_6_, beta-carotene, folic acid, niacin, pyridoxine, riboflavin, thiamin, vitamins A, C, D, and E; zinc, selenium, iron, magnesium; caffeine, and alcohol. Higher E-DII scores indicate a more proinflammatory diet, whereas lower scores indicate a more anti-inflammatory diet.

### 2.7. Statistical Analyses

Descriptive statistics were used to summarize participants’ sociodemographic characteristics across quartiles of the E-DII, as were the distribution of food group intakes across quartiles of E-DII. The mean and standard deviation (SD) was reported for continuous variables, and count and percentage were reported for categorical variables. To compare the two groups (cases and controls) an independent sample *t*-test was used for continuous variables and Chi square was used for categorical variables.

This study consisted of cases and controls matched on sex and age. Therefore, appropriate analytical statistics were used to examine the association between E-DII and schizophrenia status. In addition to matching for age and sex during the design phase, we further controlled in the analyses for body mass index, education, employment, diabetes, hypertension, and cardiovascular disease. The E-DII was analyzed both as a continuous variable and categorized by quartiles of exposure. Logistic regression was performed, and odds ratio (OR) and 95% confidence intervals (95%CI) were calculated, and significance was considered at *p*-value < 0.05. All analyses were performed using Stata 15.0 software. Quartiles of E-DII scores were calculated based on the distribution of E-DII among controls according to the following ranges: Quartile 1 ≤0.71, Quartile 2 0.71–1.78, Quartile 3 1.79–2.45 and Quartile 4 ≥2.46.

## 3. Results

The mean E-DII score for the entire Bahraini sample (cases and controls) was 1.79 ± 1.52, which indicates a pro-inflammatory diet. The cases with schizophrenia appeared to have a statistically higher E-DII score compared to their corresponding age- and sex-matched controls (1.99 ± 1.39 and 1.60 ± 1.38, respectively, *p* = 0.009). Table 1 describes the distribution of several sociodemographic and health indicators according to schizophrenia status. Cases with schizophrenia were more likely to be obese (46.7%), physically inactive (76.7%), currently smokers (61.7%), short-duration sleepers (43.3%), and have higher risks to type 2 diabetes mellitus (30.8%), hypertension (31.7%), CVD (8.3%), and muscular and joints disease (36.7%). Furthermore, cases with schizophrenia appeared to have lower socioeconomic indicators, including lower education level, and to be unemployed and single.

Supplementary Table S1 describes the distribution of the health and socio-demographic characteristics across quartiles of E-DII for the controls, while Table 2 describes the distribution of health and sociodemographic characteristics across quartiles of E-DII scores for cases with schizophrenia. According to Supplementary Table S1, Bahraini controls in the most pro-inflammatory group (i.e., quartile 4) were more likely to have higher BMIs, be physically inactive, be current smokers, and had inadequate sleep. For the patients with schizophrenia (Table 2), quartile 1 included more patients who were obese and overweight; patients in quartile 2 were mostly single, unemployed and have a higher prevalence of T2DM or hypertension; patients in quartile 3 were physically inactive and current smokers.

Supplementary Table S2 and Table 3 shows the distribution of food groups’ intake across quartiles of E-DII scores for controls and cases, respectively. Most pro-inflammatory nutrients or foods components such as energy, protein, carbohydrates, fat, saturated fats, and cholesterol, all increased linearly across the quartiles of E-DII. Likewise, anti-inflammatory dietary components such as fiber, folic acid, β-carotene, Mg, Zn, vitamins C, and A decreased across quartiles. Pro-inflammatory components that were consumed in higher quantities among subjects in the fourth quartile were protein, carbohydrates, fat, saturated fat, MUFA, PUFA, trans- fat, cholesterol, omega-6 FAs, niacin, and energy.

ORs and 95% CIs for schizophrenia status according to quartiles of the E-DII are shown in Table 4. Results obtained from modeling E-DII as a continuous variable in relation to schizophrenia suggested a positive association after adjusting for age, sex, body mass index, education, employment, diabetes, hypertension, and CVD in the analysis (OR 1.62; 95% CI 1.17–2.26). A similar association was observed when E-DII was expressed as quartiles, with participants in most pro-inflammatory group (Quartile 4) being almost six times more likely to be schizophrenic than participants in the most anti-inflammatory group (Quartile 1) (OR 5.96; 1.74–20.38; *p*-trend = 0.01).

## 4. Discussion

Underlying the relevance of this study are two related and profoundly important questions: Does inflammation precede or follow neuropsychiatric disorders (such as schizophrenia) and (2) are they causally related [40,41]. In an attempt to help answer these questions, the current study tried to examine the inflammatory potential of food intake of individuals with schizophrenia compared with their corresponding controls in the Bahraini population. The positive association observed in this study supports an association, thought the case–control design cannot comment on temporality and therefore cannot provide strong causal inference.

The presence of a significant association between schizophrenic patients and the E-DII in the current study mirrors a previous, larger-scale survey in the UK, where 254 schizophrenia patients were found to have highly elevated DII scores in comparison with disease-free controls, after adjusting for age, gender, and total energy intake [9]. Other studies have reported that higher levels of dietary inflammation were observed in schizophrenic patients: The same group that also experiences significantly worse physical health outcomes than other classes of severe mental illness [42,43].

According to the available literature and to best of our knowledge, this is the first case–control study in the Middle East and Gulf Cooperation Council (GCC) region and second in the world tackling the association between schizophrenia and dietary inflammation using the DII as a surrogate indicator for the inflammatory potential of food consumed. Relevant case–control studies on schizophrenia investigated different aspects of the inflammation–schizophrenia relationship. One study on Polish schizophrenia patients detected a functional polymorphism in the proinflammatory cytokine IL-6 and the anti-inflammatory cytokine IL-10 genes in patients with paranoid schizophrenia [44]. Such a polymorphism leads to a state of imbalance which, in turn, leads to increased levels of inflammation that predisposes to the development and progression of schizophrenia. Findings from that study were later confirmed by another case–control study, which showed that schizophrenic patients had significant increases in intracellular components of a main proinflammatory pathway, along with a substantial decrease in the anti-inflammatory ones [45].

Schizophrenia is an inflammation-related mental disorder [6,40,41,46,47,48,49,50,51,52], with variable backgrounds including biological (hormonal and genetic), lifestyle behavioral and dietary habits that predispose or accompany the development of that mental disorder [53,54].

There is mounting evidence indicating that the relationship between inflammation and mental disorders, such as schizophrenia, is a two-way relationship. While neuropsychiatric disorders (i.e., schizophrenia and mood disorders) facilitate inflammatory reactions; inflammation promotes schizophrenia and other neuropsychiatric disorders [40]. It has been shown in repeated reports that patients with neuropsychiatric disorders exhibit all classical features of inflammation, including increased activated sensors, inflammatory mediators, and circulating levels of inflammatory inducers targeting all tissues. Proinflammatory cytokines modulate cognition and mood behavior by inhibiting neuroendocrine responses, lowering brain monoamine levels, impairing brain plasticity, and promoting excitotoxicity (increased glutamate levels) [40]. The important triggering factors of inflammation that predispose to developing schizophrenia include changes in neuroendocrine regulation [55,56], metabolism [6,57,58,59], diet/microbiota [60,61,62,63,64], and adverse health behaviors such as smoking [65,66,67,68]. Finally, recent reports indicate that early-life stress is associated with inflammation before the progression of neuropsychiatric disorders such as schizophrenia [69,70].

Considering that the DII is as a sensitive index for six inflammatory markers: CRP, IL-1β, IL-4, IL-6, IL-10, and TNF-α [31]; it can be inferred that lowering the intake of pro-inflammatory, high-DII foods and increasing the consumption of anti-inflammatory, low-DII foods would act as powerful adjuvant therapy for schizophrenia patients [71]. This conclusion is reinforced by the fact that the aforementioned pro-inflammatory cytokines are those mediators that are directly involved in the pathogenesis of schizophrenia and its predisposed and associated elevated inflammatory state [47,49,65,67,72,73,74]. Further, other macro- and micro-nutrients involved in the calculation of DII are also involved in maintaining healthy brain function and improving neuroplasticity, and thus nullify the progression of schizophrenia or hinder its severity in diagnosed patients [51]. These nutrients include omega-3 polyunsaturated fatty acids, vitamin D, B vitamins (B_6_, folate, B_12_), vitamin E, and carotenoids [51].

Findings of the current study are consistent and in line with the general context regarding the relationship between schizophrenia and inflammation. Our results are supported by cross-sectional studies showing that individuals with severe mental illnesses, particularly schizophrenia, consume more pro-inflammatory foods and fewer anti-inflammatory nutrients than the general population [8,9].

This study has several strengths. It is the first study in Bahrain and Middle East to explore the association between the inflammatory properties of the diet in relation to schizophrenia. Second, the matched case–control design that allows for a relatively large sample resulting in relatively high statistical power and the ability to control for several covariates. Third, the use of the DII as a research tool allowed us to examine the overall diet rather than focusing on individual nutrients or foods. Fourth, E-DII scores were computed using an FFQ that was specifically designed to capture the broad range of diet in Bahrain. Despite its strengths, the study also has limitations, including the possibility of both selection and information biases. Another limitation is that DII/ E-DII was not validated with inflammatory markers in this study. Though it would have been ideal if we had information on inflammatory markers, the study had already been conducted and the current analyses were dependent on existing data. Thus, it was not be possible to perform validation analyses. To countervailed this concern, however, it is important to note that the DII or E-DII have been validated in over 20 studies with various inflammatory markers across the world, including Middle Eastern countries [33,34,75]. On balance, the current study adds to the existing evidence that schizophrenia is associated with increased exposure to pro-inflammatory foods, as expressed by the DII/ E-DII. Future longitudinal studies are recommended to assess how dietary inflammation is related to the onset of schizophrenia, as no reliable evidence linking dietary inflammation with the risk of schizophrenia currently exists.

Future studies are encouraged to account for the duration and severity of mental illness using well-known tools such as the Positive and Negative Syndrome Scale (PANSS) as a measure for illness severity.

From the mental health practice perspectives, the patients’ assessment of quality of diet and specifically the inflammatory properties of food appear to be an important practice in the care of individuals with schizophrenia. This research raises the recommendation that patients with schizophrenia should be regularly evaluated for dietary and lifestyle factors in order to improve the outcomes of the illness.

From research perspectives, follow-up studies that would measure both DII and concentrations of inflammatory cytokines in plasma, serum, and other body fluids are encouraged to better understand the relationship between diet and schizophrenia. Furthermore, the effectiveness of anti-inflammatory diet in the management of schizophrenia needs to be examined using randomized controlled clinical trials. 

## 5. Conclusions

The current study shows that individuals with schizophrenia consume a more pro-inflammatory diet, with fewer anti-inflammatory nutrients, than the general population. This, in turn, would imply that instead of only changing the caloric intake of patients or the quality of the diet, researchers and clinicians need to take into consideration the inflammatory role of the diet of patients with schizophrenia.

## Figures and Tables

**Table 1:** Socio-demographic characteristics of schizophrenia cases and healthy controls.

Characteristic Variable	Cases	Controls	*p*-Value
**Continuous variables** (mean ± SD)			
Age (years)	41.69 ± 13.00	41.63 ± 13.24	0.96
Weight (Kg)	83.58 ± 23.05	77.43 ± 17.02	0.02 *
Height (cm)	165.39 ± 9.01	166.98 ± 9.61	0.19
Body mass index (BMI) (Kg/m^2^)	30.65 ± 9.24	27.58 ± 6.25	0.003 *
**Categorical variables:**			
Male (%)	55	55	-
Overweight (%)	23.33	37.27	0.40
Obese (%)	46.67	27.73	0.002 *
Education level (%)			
Primary or less	36.7	10.8	0.001 *
Secondary	43.3	30.0	
Tertiary	20.0	59.2	
Job (%)			
Un-employed	43.3	0.8	0.001 *
Employed/Student	39.2	85.0	
Retired	17.5	14.2	
Marital status (%)			
Married/Engaged	30.00	79.17	0.001*
Single	55.00	14.17	
Divorced	14.17	3.33	
Widow/er	0.83	3.33	
Physical activity level (%)			
Inactive	76.67	40.00	0.001 *
Active	23.33	60.00	
Current smoking status (%)			
Nonsmoker	61.67	85.83	*
Smoker	38.33	14.17	
Smoking type (%)			
Cigarettes	58.70	64.71	0.001 *
Water Pipe	4.35	11.76	
Mixed	36.95	17.65	
Electronic Cigarette	0.0	5.88	
Medical comorbidity (%)			
Type 2 Diabetes mellitus	30.83	10.00	0.001 *
Hypertension	31.67	14.17	0.001 *
Cardio vascular disease	8.33	5.83	0.45
Muscular & joints disease	36.67	23.33	0.02 *
Adequate Sleep (%)	56.67	92.44	0.001 *

* Cases were significantly different from controls at *p* < 0.05.

**Table 2:** Characteristics of schizophrenia case participants across the quartiles of energy-adjusted Dietary Inflammatory Index (E-DII).

Characteristic	Quartile 1	Quartile 2	Quartile 3	Quartile 4
Age (years): Mean ± SD	46.44 ± 15.00	41.25 ± 14.16	43.66 ± 12.51	37.56 ± 10.00
Body mass index (BMI) (Kg/m^2^): Mean ± SD	30.69 ± 6.64	30.64 ± 9.47	31.10 ± 11.90	30.28 ± 8.12
Overweight (%)	31.81	19.36	23.33	21.62
Obese (%)	54.55	45.16	46.67	43.24
Education (%)				
Primary	26.7	40	41.9	37.9
Secondary	56.7	20	41.9	34.5
Tertiary	16.7	20	16.1	27.6
Job (%)				
Unemployed	40	50	38.7	44.8
Employed/Student	43.3	40	32.3	41.4
Retired	16.7	10	29	13.8
Social Status (%)				
Married/Engaged	30	26.7	45.2	17.2
Single	50	63.3	38.7	69
Divorced	20	6.7	16.1	13.8
Widow/er	0	3.3	0	0
Physical activity (%):				
Inactive	73.3	76.7	83.9	72.4
Active	26.7	23.3	16.1	27.6
Smoking (%)				
Nonsmoker	36.3	66.7	58.1	58.6
Smoker	36.7	33.3	41.9	41.4
Smoking Type (%)				
Cigarette	54.5	60	76.9	41.7
Water Pipe	9.1	10	0	0
Mixed	36.4	30	23.1	58.3
Medical Comorbidity (%)				
Type 2 Diabetes mellitus	36.7	36.7	32.3	17.2
Hypertension	33.3	36.7	32.3	24.1
Cardiovascular disease	6.7	13.3	6.5	6.9
Muscular & joints disease	36.7	43.3	22.6	44.8
Adequate Sleep (%)	50	66.7	45.2	65.5

**Table 3:** Distribution of nutrients and dietary factors across quartiles of energy adjusted Dietary Inflammatory Index (E-DII) for schizophrenia cases.

Nutrient or Dietary Factor/Day (Mean ± SD)	Quartile 1	Quartile 2	Quartile 3	Quartile 4
Energy (Kcal)	2113.72 ± 512.3	2715.78 ± 832.29	2469.4 ± 814.44	2856.51 ± 810.95
Protein (g)	86.64 ± 20.72	115.87 ± 38.62	105.37 ± 30.31	116.29 ± 29.86
Carbohydrates (g)	310.06 ± 71.52	372.53 ± 117.92	322.52 ± 104.97	364.62 ± 109.32
Fibers (g)	26.53 ± 10.86	29.01 ± 13	20.55 ± 8.05	22.9 ± 9.75
Fat (g)	63.01 ± 21.31	90.49 ± 33.22	88.79 ± 35.98	108.11 ± 37.02
SFA (g)	22.94 ± 7.09	33.54 ± 10.94	31.39 ± 10.45	40.13 ± 13.02
MUFA (g)	8.18 ± 3.49	11.58 ± 6.12	12.96 ± 11	12.29 ± 6.1
PUFA (g)	9.06 ± 4.56	11.43 ± 5.7	13.25 ± 9.7	12.84 ± 5.4
Trans fats (g)	0.37 ± 0.24	0.43 ± 0.25	0.29 ± 0.16	0.37 ± 0.21
Cholesterol (mg)	372.7 ± 217.79	579.4 ± 300	501.72 ± 200.86	607.58 ± 248.07
Omega-3 (g)	0.59 ± 0.2	0.81 ± 0.34	0.93 ± 0.72	0.75 ± 0.38
Omega-6 (g)	7.55 ± 4.03	9.41 ± 4.81	11.29 ± 8.92	10.71 ± 4.63
Thiamin (mg)	1.29 ± 0.32	1.55 ± 0.49	1.45 ± 0.74	1.34 ± 0.4
Riboflavin (mg)	1.12 ± 0.35	1.54 ± 0.69	1.37 ± 0.8	1.27 ± 0.62
Niacin (mg)	16.02 ± 6.97	19.39 ± 9.59	17.32 ± 9.64	18.42 ± 7.43
Vitamin B_6_ (mg)	0.96 ± 0.29	1.09 ± 0.39	0.97 ± 0.65	0.87 ± 0.36
Vitamin B_12_ (µg)	1.88 ± 0.82	2.97 ± 1.8	3.41 ± 2.73	2.8 ± 2.31
Vitamin C (mg)	125.03 ± 44.08	105.75 ± 38.17	90.56 ± 37.39	88.09 ± 43.76
Vitamin D (IU)	64.86 ± 52.43	94.22 ± 72.07	76.7 ± 60.99	58.63 ± 59.94
Vitamin E (mg)	2.63 ± 1.1	3.13 ± 1.64	2.86 ± 1.85	3.11 ± 1.69
Folic Acid (µg)	458.82 ± 225.73	534.7 ± 212.28	373.91 ± 165.98	350.11 ± 129.41
Vitamin A (IU)	6472.23 ± 1953.38	6412.42 ± 2022.39	5066.16 ± 1980.85	4543.75 ± 1968.46
β-carotene (µg)	2737.61 ± 931.49	2455.61 ± 900.81	1966.68 ± 926.29	1527.49 ± 871.88
Iron (mg)	14.67 ± 4.73	17.76 ± 6.27	15.58 ± 7.91	14.94 ± 4.84
Magnesium (mg)	225.4 ± 98.21	249.91 ± 128.36	179.48 ± 77.05	199 ± 76.42
Selenium (µg)	55.73 ± 16.38	69.45 ± 25.69	63.76 ± 31.95	60.93 ± 17.07
Zinc (mg)	4.96 ± 1.57	6.4 ± 2.86	5.3 ± 2.58	5.07 ± 2.52
Alcohol (g)	0.05 ± 0.23	0 ± 0	0.26 ± 1.26	0.97 ± 2.82
Caffeine (mg)	179.51 ± 260.16	190.2 ± 208.82	95.38 ± 121.05	168.7 ± 171.97

**Table 4:** Odds ratios (OR) and confidence intervals for quartiles of energy adjusted Dietary Inflammatory Index (E-DII) associated with the diet of patients with Schizophrenia.

	Quartiles of the Energy-Adjusted Dietary Inflammatory Index *				
	Quartile 1	Quartile 2	Quartile 3	Quartile 4	E-DII (Continuous)
E-DII Range	≤0.07	0.71–1.78	1.79–2.45	≥2.46	
Cases/Controls	11/30	38/30	28/30	43/30	120/120
Multivariate-adjusted ^a^	1 (Ref)	4.27 (1.27–14.35)	2.78 (0.77–10.0)	5.96 (1.74–20.38)	1.62 (1.17–2.26)

* *p* trend = 0.01. ^a^ Adjusted for age, sex, body mass index, education, employment, diabetes, hypertension, and cardiovascular disease.

## Supplementary Materials

The following are available online at https://www.mdpi.com/2072-6643/11/8/1867/s1, Table S1, Characteristics of control participants across the quartiles of energy adjusted Dietary Inflammatory Index (E-DII), Table S2, Distribution of nutrients and dietary factors across quartiles of energy adjusted Dietary Inflammatory Index (E-DII) for controls.

## Disclaimer

James R. Hébert owns controlling interest in Connecting Health Innovations LLC (CHI), a company that has licensed the right to his invention of the dietary inflammatory index (DII®) from the University of South Carolina in order to develop computer and smart phone applications for patient counseling and dietary intervention in clinical settings. Nitin Shivappa is an employee of CHI. The subject matter of this paper will not have any direct bearing on that work, nor has that activity exerted any influence on this project.
