# Supplementary material for: Increased Dietary Inflammatory Index Is Associated with Schizophrenia: Results of a Case–Control Study from Bahrain
Source: Nutrients. 2019 Aug 11;11(8):1867. doi: 10.3390/nu11081867 (PMC6722742; doi:10.3390/nu11081867)
Supplement: Supplementary file 1 [file nutrients-11-01867-s001.pdf]

Demographics

Research ID#

Sex: ☐Male ☐Female
Age (years): ……..
Education level: ☐Illiterate ☐Reads and Write ☐Primary/Intermediate School level ☐Secondary School level ☐Diploma ☐Bachelor level ☐MSc./MBA/MA ☐Doctorate Level
Occupation: ☐Unemployed ☐Self-Employed Person ☐Employee ☐Civil Servant ☐University Student ☐Pensioner/Retired ☐Housewife
Marital Status: ☐Married/Engaged ☐Single ☐Divorced ☐Widow/er
Income: ☐≤250 BD ☐251-499 BD ☐500-999 BD ☐+1000BD
Governorate: ☐Capital ☐Northern ☐Muharraq ☐Southern

General Health
Measure Weight …….Kg and Height ……centimeter

Do you suffer from the following diseases?

Diabetes mellitus ☐Yes ☐No
Hypertension ☐Yes ☐No
Cardiovascular problems ☐Yes ☐No
Muscular & Joints problems ☐Yes ☐No
Epilepsy ☐Yes ☐No

Dehydration - Do you have the following symptoms? Over the past 7 days

Chills/fever ☐Yes ☐No
Constipation ☐Yes ☐No
Diarrhea ☐Yes ☐No
Lack of Concentration ☐Yes ☐No
Lack of Energy ☐Yes ☐No
Gas or indigestion ☐Yes ☐No
Skin problems e.g. dry, allergies ☐Yes ☐No
Dull hair, split ends ☐Yes ☐No
Nails broken easily ☐Yes ☐No
Frequent Colds/Flus ☐Yes ☐No
Frequent Headaches ☐Yes ☐No

Psychiatric History

Diagnosis: ☐Schizophrenia (Any type) ☐Bipolar Affective Dis. ☐Major Depression Dis.
Time of Diagnosis: ☐<6 month ☐6-12 months ☐13-24 months ☐≥25 months
Number of OPD Visits (in the past 12 months): ☐0 ☐1 ☐2 ☐3 ☐4 ☐5 ☐6 ☐7 ☐8 ☐≥9
Number of Admissions (Since Diagnosis): ☐0 ☐1 ☐2 ☐3 ☐4 ☐5 ☐6 ☐7 ☐8 ☐≥9

Life Style

Physical Activity – IPAQ Based

Think about all the physical activities that you did in the **last 7 days**. Think **only** about those physical activities that you did for **at least** **10 minutes at a time.**

Activity Level	*Frequency (days per week)*	*Duration (minutes)*
**Vigorous activities** e.g. heavy lifting, digging, aerobics, or fast bicycling		
**Moderate activities** e.g. carrying light loads, bicycling at a regular pace or double tennis		
**Walking** (Low Intensity)		
**Sitting** (Complete Rest) e.g. TV		

Work Pattern

How many hours per day usually do you work? …..Hrs
What is your work pattern? ☐Regular (morning only) ☐Shifts (morning/noon/night)
What is the usual area of your work? ☐Indoors mainly ☐Outdoors mainly

Sleeping pattern

How many hours do you usually sleep per day?
☐Less than 4 Hrs ☐4-6 Hrs ☐7- 8 Hrs ☐More than 8 Hrs

Do you usually smoke?

☐No
☐Yes, please indicate what type (you tick more than one)?
☐Cigarettes ☐Shisha (Hooka) ☐Electronic Shisha ☐Mixed

Food Intake

How many main meals do you take per day?
☐Less than 3 meals/day ☐3 meals per day ☐More than 3 meals/day

**Mark HOW OFTEN you consumed the following foods during the last month:**<br>**Caution,** answer considering as portion the quantity that is entered in the parenthesis.<br>(*t* =times, *g*= gram, pcs =pieces, *c* = cup =240 ml)						
	**Never/ Rarely**	**1-3 t/ month**	**1-2 t/ week**	**3-6 t/ week**	**1 t/ day**	**≥ 2 t/ day**
White "Lebanese/Arabic" bread (1 piece 30gr)						
White toasted bread (2 pcs)						
Brown "Lebanese/Arabic" bread (1 slice 30gr)						
Brown toasted grain bread (2 pcs)						
Biscuits (2 pcs), rusks (1 pcs), cookies (2 pcs)						
Cereals (1 cup), cereals bar (1 pcs)						
Beef (steak) (1p~150 grams)						
Burgers (1 pc), meatballs (4 pcs), minced meat (1c)						
Chicken (all types) (150 gr)						
Lamb, goat, deer, rabbit, lamb chops (150 grams)						
Fish (approx. 150 g)						
Seafood (octopus, squid, shrimp) (150 grams)						
Lentils, beans, chickpeas (1 c) (1 dish = 2 cups)						
Fresh soup, (1 portion=250ml)						
Soup with pasta (e.g. noodles) (1 portion=250ml)						
Rice, (1 cup, 1 medium plate)						
Boiled potatoes, mashed potatoes (1 medium)						
French fries (1 portion)						
Fresh Vegetables (any kind)						
Fresh Fruits (any kind)						
Dried fruits (¼ cup)						
Dried nuts, nuts (¼ cup)						
Yoghurt complete or light (1 tub)						
Cream cheese "Glasses" (25 gr)						
Feta, white cheese, hard cheese (25 gr)						
Egg (boiled, fried, omelet) (1 pcs)						
Pies (ex. Cheese pie, spinach pie) (1 portion)						
Ice cream, milk shake, pudding, rice pudding (1 pcs)						
Honey, jam (1 teaspoon)						
Olives (10 small /5 large)						
Chocolate (all types) (1 medium = 60 gr)						
Chips packs, popcorn (1 bag =70 gr)						

Fluid Intake

How many water glasses (water bottles) do you usually take per day (24 Hours)?

☐None ☐1 ☐2 ☐3 ☐4 ☐5 ☐6 ☐7 ☐8 ☐9 ☐10 ☐more than 10

Do you drink water overnight?

☐Never/Rarely ☐1-2 t/week ☐3-6 t/week ☐1-2 t/ day ☐3-4 t/day ☐>5 t/day

What kind of water do you usually take at home?

☐Tap water ☐Barreled water ☐Bottled water ☐Filtrated water

**Mark HOW OFTEN you consumed the following fluids during the last month**<br>**Caution**, answer considering as portion the quantity that is entered in the parenthesis						
	**Never/ Rarely**	**1-2 t/ week**	**3-6 t/ week**	**1-2 t/ day**	**3-4 t/ day**	**>5 t/ day**
Fruit juice (1 glass or 1 small juice pack)						
Soft drinks, (1 can)						
Milk, milk shake (1 glass)						
Coffee in a cup (e.g. Americano, Espresso)						
Arabic Coffee						
Tea, other herbal teas (e.g. chamomile, peppermint) (1 cup)						
Isotonic/energy drinks (1 glass)						
Alcoholic drinks (wine, beer, whisky, vodka, (1 unit)						

Elimination of body fluids

The quantity of your body sweat expulsion **during exercise** from 1 (minimum) until the 10 (maximum) corresponds to:

☐1 ☐2 ☐3 ☐4 ☐5 ☐6 ☐7 ☐8 ☐9 ☐10

The quantity of your body sweat expulsion **in regular conditions** from 1 (minimum) until the 10 (maximum) corresponds to:

☐1 ☐2 ☐3 ☐4 ☐5 ☐6 ☐7 ☐8 ☐9 ☐10

The expulsion of urine from your body corresponds to:

☐1t/day ☐2-4t/day ☐5-7t/day ☐8-10t/day ☐more than 10t/day

The expulsion of faeces from your body corresponds to:

☐≥1t/day ☐5-6t/ week ☐3-4t/ week☐1-2t/ week ☐1t/10days

Supplementary Table S1. Characteristics of control participants across the quartiles of energy adjusted Dietary Inflammatory Index (E-DII).

**Characteristic**	**Quartile 1**	**Quartile 2**	**Quartile 3**	**Quartile 4**
Age (years): Mean ± SD	45.25±13.57	42.17±14.21	36.48±10.75	38.17±11.15
Body mass index (BMI) (Kg/m^2): Mean ± SD	27.54±5.04	28.53±7.19	25.38±4.65	29.69±8.04
Overweight (%)	35.89	38.23	40	34.78
Obese (%)	28.21	29.63	16.67	39.13
Education (%)				
• Primary	16.7	20	6.7	0
• Secondary	23.3	26.7	33.3	36.7
• Tertiary	60	53.3	60	63.3
Job (%)				
• Unemployed	3.3	0	0	0
• Employed/Student	66.7	83.3	93.3	96.7
• Retired	30	16.7	6.7	3.3
Social Status (%)				
• Married/Engaged	80	76.7	73.3	86.7
• Single	10	13.3	23.3	10
• Divorced	0	6.7	3.3	3.3
• Widow/er	10	3.3	0	0
Physical activity (%):				
• Inactive	40	40	30	50
• Active	60	60	70	50
Smoking (%)				
• Nonsmoker	90	93.3	76.7	83.3
• Smoker	10	6.7	23.3	16.7
Smoking Type (%)				
• Cigarette	100	50	57.1	60
• Water Pipe	0	0	14.3	20
	0	0	14.3	0
• Mixed	0	50	14.3	20
Medical Comorbidity (%)				
• Type 2 Diabetes mellitus	10	13.3	10	6.7
• Hypertension	16.7	20	10	10
• Cardiovascular disease	0	10	10	3.3
• Muscular & joints disease	30	30	16.7	16.7
Adequate Sleep (%)	93.3	90	93.1	93.3

Supplementary Table S2. Distribution of nutrients and dietary factors across quartiles of energy adjusted Dietary Inflammatory Index (E-DII) for controls.

Nutrient or Dietary Factor/Day (Mean ± SD)	Quartile 1	Quartile 2	Quartile 3	Quartile 4
Energy (Kcal)	2024.75±579.46	2174.98±638.32	2636.62±1018.52	2911.19±1032.99
Protein (g)	87.79±25.77	99.37±40.97	114.54±51.48	131.73±52.67
Carbohydrates (g)	283.03±83.06	296.63±91.57	339.46±125.85	353.93±137.14
Fibers (g)	27.35±16.19	20.22±7.67	26.07±15.11	23.8±11.93
Fat (g)	64.99±21.89	70.33±28.4	97.39±42.35	113.58±41.69
SFA (g)	22.37±6.33	25.7±9.7	36.2±15.82	41.61±16.82
MUFA (g)	9.13±2.63	9.83±4.8	10.56±6.27	14.08±9.19
PUFA (g)	10.1±4.17	10.44±6.87	10.59±5.68	12.83±6.57
Trans fats (g)	0.29±0.15	0.44±0.28	0.39±0.29	0.55±0.54
Cholesterol (mg)	327±145.18	420.3±227.06	529.62±395.93	605.3±318.99
Omega-3 (g)	0.69±0.26	0.76±0.35	0.67±0.4	0.8±0.42
Omega-6 (g)	8.18±3.16	8.71±5.61	8.57±4.61	10.49±5.57
Thiamin (mg)	1.36±0.47	1.29±0.47	1.3±0.7	1.39±0.45
Riboflavin (mg)	1.39±0.53	1.54±0.74	1.49±0.74	1.4±0.6
Niacin (mg)	18.12±7.89	18.71±8.49	20.55±10.8	20.11±8.74
Vitamin B_6 (mg)	1.13±0.54	1.12±0.7	1.01±0.6	0.97±0.44
Vitamin B_{12} (μg)	2.91±1.54	3.49±2.25	2.96±2.21	3.39±2.23
Vitamin C (mg)	92.06±34.12	76.04±30.73	87.61±35.55	86.58±40.05
Vitamin D (IU)	94.04±55.85	110.03±76	81.75±59.13	62.4±75.3
Vitamin E (mg)	2.34±0.9	2.33±1.07	2.78±1.6	3.28±2.13
Folic Acid (μg)	5315.05±1915.16	4394.31±1686.85	4802.14±1982.45	5065.32±2373.3
Vitamin A (IU)	2072.71±840.09	1567.65±760.28	1695.51±717.28	1766.35±945.72
β-carotene (μg)	485.68±285.73	377.67±146.2	420.45±261.42	360.68±166.38
Iron (mg)	16.28±6.92	14.83±6.15	15.66±8.29	16.27±5.92
Magnesium (mg)	254.56±114.86	223.21±98.74	270.56±145.85	232.56±104.31
Selenium (μg)	57.52±19.8	56.94±14.24	55.54±29.88	57.9±22.92
Zinc (mg)	5.62±2.19	4.66±1.96	5.41±3.46	5.54±3.2
Alcohol (g)	0±0	0±0	0±0	0±0
Caffeine (mg)	210.94±295.64	237.01±231.54	324.73±307.88	226.16±237.24
